# Gut Biome and Mental Health: Do Probiotics Work?

**DOI:** 10.7759/cureus.40293

**Published:** 2023-06-12

**Authors:** Jayakrishna S Madabushi, Priyal Khurana, Nihit Gupta, Mayank Gupta

**Affiliations:** 1 Psychiatry, Alabama College of Osteopathic Medicine, Birmingham, USA; 2 Psychology, Christ University, Jaipur, IND; 3 Psychiatry, Dayton Children’s Hospital, Dayton, USA; 4 Psychiatry and Behavioral Sciences, Southwood Psychiatric Hospital, Pittsburgh, USA

**Keywords:** psychobiotics, mental health, gut-brain axis, depression, anxiety

## Abstract

Mental health conditions have been linked closely to an imbalance of microbiota in the gut, leading to disruption of the microbiome (dysbiosis). Several neurotransmitters, such as GABA (gamma-aminobutyric acid), serotonin, and glutamate, are produced in the gut, which are associated with anxiety and depressive symptoms. Mental health and the gut have been linked closely, and many mental illnesses have been associated with gut dysbiosis. Probiotics are marketed to improve gut health, act as mood enhancers, and be effective in reducing stress as unregulated over-the-counter supplements. Given healthcare disparities and patient-doctor gaps across the globe, this review aims to appraise the literature on probiotics for the prevention and treatment of mental disorders. PubMed and Google Scholar databases were searched till March 2023 using the MeSH words “prebiotics,” “probiotics,” “synbiotics,” and “psychobiotics.” Out of 207 studies, 26 studies met the inclusion criteria and were included in the review. Studies suggest probiotics could be an effective and economical adjunct therapy; however, due to weak study design and low power, the results are inconclusive. Their use is not without risks, and healthcare providers need close supervision until more robust longitudinal studies are conducted to appraise their efficacy and safety profiles.

## Introduction and background

The global burden of mental disorders continues to mount, with the sixth leading cause for the number of years of a person’s life lost due to health-related conditions, referred to as disability-adjusted life years (DALYs) [1]. The Anxiety and Depression Association of America quoted depression to be the most prevalent psychiatric condition affecting nearly 264 million people worldwide, and approximately half of those are diagnosed with anxiety [2]. A survey of 150,000 adults from 26 countries reported a 3.7% lifetime prevalence of anxiety disorders [3]. The psychiatrist per population in the developed versus the developing world is staggeringly low. In a recent survey of 7,870 individuals with depression from 49 countries, only 32% had a formal diagnosis, and only a few ever received treatment [4,5]. Approximately 75% of individuals with mental health conditions in developing nations remain untreated, and almost a million people lose their lives to suicide each year [6]. Since the outbreak of COVID-19, the global prevalence of depression, initially estimated to be 3.44% in 2017, has increased about sevenfold [7].

The term “psychobiotic” was introduced for beneficial live bacteria (probiotics) or other compounds (prebiotics, synbiotics) that may influence gut neurotransmitters [8]. Studies have investigated their effects on psychiatric disorders, with mixed findings [9]. They are widely consumed as over-the-counter supplements, which are unregulated but extensively marketed products with claims of effectiveness for mental health conditions. Even though there is a lack of objective cost-benefit analysis, their use is prevalent in individuals who do not seek psychiatric help, lack access to mental health care, or cannot afford it. These conundrums inspired further systematic inquiries about its utility in marginalized low-income households with poor access to mental health care and developing nations with a lower ratio of psychiatrists to patients. 

The theoretical model is based on decades of research on the human digestive tract, which houses tens of trillions of microorganisms, including various bacteria, viruses, phages, protozoa, fungi, and archaea. This is referred to as the human gut microbiome [10]. These microbes inhabiting the human gut typically outnumber the total number of human cells. This is true for the human DNA in the body, too; the ratio between human and bacterial genes is approximately 1:100 [11]. Recent studies have proposed that extensive diversity of the gut microbiota influences most physiological processes ranging from metabolic diseases to mental health and, therefore, potential for impact on mood and cognition [12]. In 2007, the Human Microbiome Project was initiated, followed by the Human Microbiome Project Consortium and METAgenomics of Human Intestinal Tract (MetaHIT) [13], to investigate these initial findings. The gut and brain communicate through complex bidirectional pathways, including the enteric nervous system (also called the second brain), autonomic nervous system, and neuroendocrine and immune pathways [14]. A recent study proposed another plausible pathway of visceral-fugal neurons, which can sense the happenings inside the gut wall and communicate this sensory information to other organs, such as the brain and spinal cord, which subsequently impacts cognition, mood, and general well-being [15]. Most neurotransmitters are also produced in the gut [16]. Approximately 50% of dopamine and 95% of serotonin is produced in the gastrointestinal (GI) tract. The widely known hypothalamic-pituitary-adrenal (HPA) axis is postulated to get activated with a disruption in the gut microbiota [17]. Early studies have shown that when the microbiome is diverse, it also helps the HPA axis to regulate and deal efficiently with external stressors in desperate times. In contrast, when the gut microbiome is less diversified, it is easily disturbed and falls out of equilibrium in times of stress. The gut then enters a dysbiotic state, which can make the intestine's lining permeable/leaky. The incompletely digested food materials can then enter the bloodstream, and this disruption in the equilibrium can cause inflammatory diseases, and recent associations have also been confirmed with mental health diseases [18-20]. Additional evidence has suggested that symptoms of depression and inflammatory gut worsen with poor food choices and lack of physical exercise [21].

Furthermore, repeated activation of the HPA axis habituating the organism with the stressors has been linked to serious health conditions such as type 2 diabetes and cardiovascular diseases [22,23]. Serotonin, dopamine, gamma-aminobutyric acid (GABA), histamine, and dozens of others have been implicated in the pathophysiology of anxiety and depression [24,25]. The majority of modern antidepressants enhance these neurotransmission networks [26,27], and, therefore, by monitoring and balancing the levels of neurotransmitters, the symptoms of depression, anxiety, and other mental health conditions could be treated [28].

The close relationship between the gut and brain, based on emerging research, indicates the role of gut dysbiosis in many mental disorders. Studies suggest that peptic ulceration and irritable bowel syndrome (IBS) are the most common GI problems co-occurring with mental health problems [29]. Another study found an undiversified microbiome in the depressed group, mostly colonizing Firmicutes, Bacteroides, and Actinobacteria in the gut [30]. 

Therefore, in this review, we appraise the literature to establish evidence to support its effectiveness for mental disorders.

## Review

Methods

**Figure 1:**
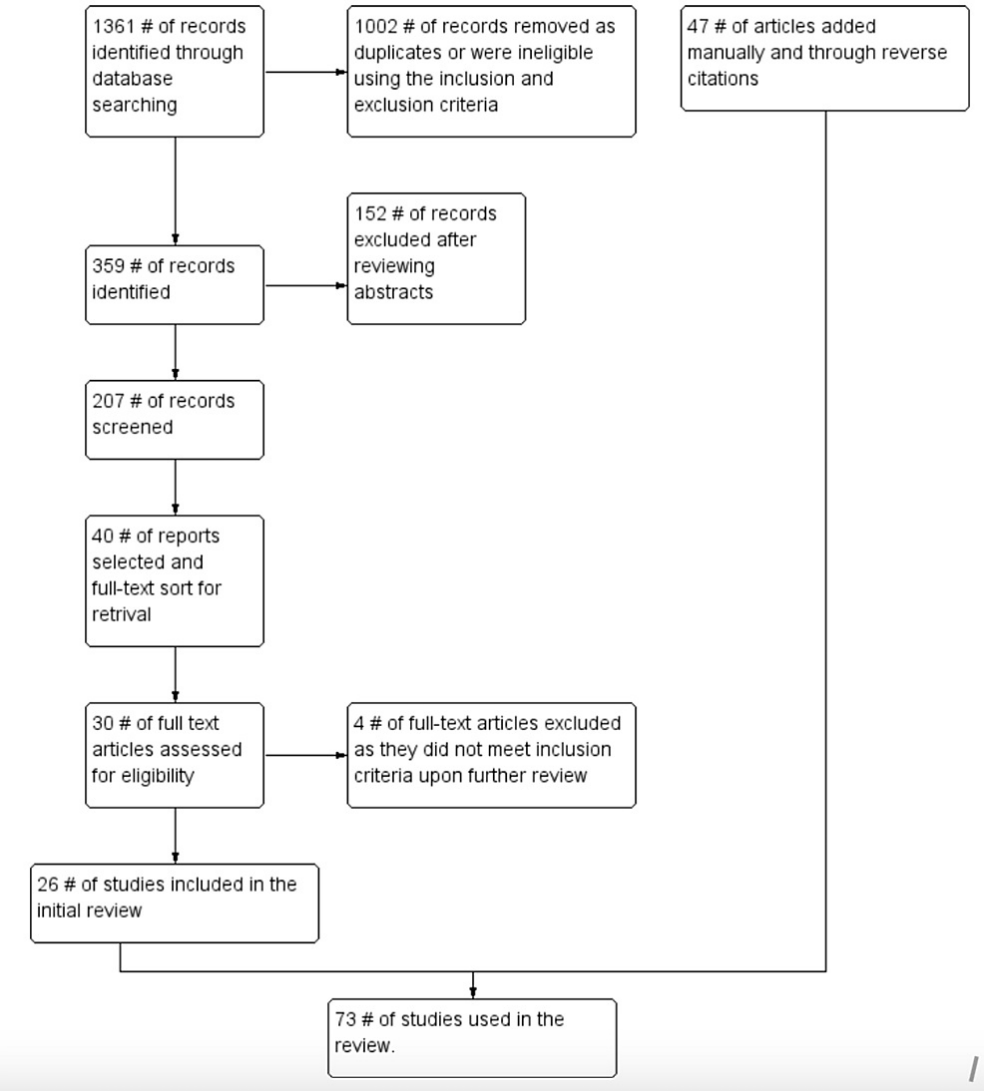
The search methodology.

Literature was searched on PubMed, Embase, and Google Scholar, and studies published till May 2021 were included. The search was conducted by P.K. and M.G. using the keywords “prebiotics,” “probiotics,” “synbiotics,” “psychobiotics,” “depression,” “anxiety,” “stress,” and “gut-brain-axis.” The references of all the relevant articles were further searched. The inclusion criteria were as follows: (1) only human studies; (2) studies available in the English language; (3) studies/interventions involving prebiotics, probiotics, synbiotics, and psychobiotics; and (4) studies that emphasized depression, anxiety, and stress. The exclusion criteria followed were as follows: (1) systematic reviews, meta-analyses, reviews, conference abstracts, commentaries, editorials, mathematical modeling studies, surveillance reports, recommendations, guidelines, and economic analyses; (2) studies dealing with general or other specific mental disorders; and (3) studies in which stress, depression, and anxiety co-occurred with other health conditions such as pregnancy, diabetes, or cardiovascular problems. In cases of disagreement, only those articles that were mutually agreed upon were included. The complete search strategy is included in Figure 1. The search yielded 207 results, of which 26 studies befitted the inclusion criteria and were included in the review.

Results and Discussion 

Anxiety Disorders 

Psychobiotics may ameliorate the effects of stress, based on intervention with a probiotic multivitamin on individuals with a borderline range of stress measured on the patient neurotoxicity questionnaire. The participants also completed a list of adjectives every two months for six months and measured the severity of GI conditions and infections, which are strong stress indicators, and scores were categorized based on positive and negative conditions. The participants taking one capsule of probiotic multivitamin of *Lactobacillus* strains and *Bifidobacterium* for six months showed improved general condition by 40%. Additionally, the positive symptoms had a mean increase of 17.4%, and the negative conditions had a mean decrease of 23.3% [31]. 

Stress also affects the immune system and stressors that are responsible for the fight or flight response; when the stressor becomes chronic, it impacts the immune system in a destructive pattern [32]. The effect of a probiotic diet on examination stress showed no significant differences in the Spielberger State-Trait Anxiety Inventory (STAI) scores between *Lactobacillus* every day and skimmed milk; however, a significant change in lymphocyte and CD56+ cell concentrations was reported. The probiotic group showed stable cortisol levels, while they were elevated in the placebo group. Therefore, fermented milk could help maintain cortisol levels in stressful times and lower infections [33]. The study does not report the role of probiotics in improving sleep and psychological symptoms in stressed individuals; however, GI symptoms of nausea, vomiting, and abdominal pain significantly decreased [34]. Another study aimed at investigating the effects of a probiotic fiber on acute stress responses, cognitive functions, and inflammation found no correlation between probiotic supplements and inflammatory profiles or psychological outcomes. However, the neuropsychological assessment revealed improved cognitive flexibility and sustained attention after the intervention, and participants in the probiotic cohort made fewer errors and completed the tasks in reduced trials [35]. 

A study reports a significant reduction in anxiety upon probiotic consumption in patients with laryngeal cancer, with stable corticotropin-releasing factor levels and heart rate compared to the placebo group [36]. The relation between probiotic consumption and altered cytokine balance in people diagnosed with generalized anxiety disorder has also been reported [37]. 

Several genes are associated with anxiety [38], including the interleukin (IL)-1β gene [39]. An Italian study reported the role of the IL-1β gene in developing anxiety and the effects of psychobiotics in reducing anxiety symptoms, with 43.24% of anxious subjects being A allele carriers of the IL-1β gene and the rest 11.43% of subjects being anxious non-carriers. The study reported decreased anxiety scores in the psychobiotic group compared with the placebo group. Additionally, anxiety symptoms can improve in healthy adults where the IL-1β gene is a risk factor [40].

Depression

In 2004, Logan and Katzman first proposed probiotics as an adjuvant treatment for depression [41]. Probiotics are reported to decrease depression scores significantly, serum levels of insulin, oxidative stress, and high-sensitivity C-reactive protein concentrations, suggesting their promising effects in treating depressive symptoms [42]. In contrast, a double-blind, randomized, controlled study by Romijn et al. evaluated the impact of probiotics on mood, anxiety, GI symptoms, stress, vitamin D levels, brain-derived neurotrophic factor (BDNF) levels, and pro-inflammatory cytokine levels. It reported no significant improvement in stress, anxiety, or mood. The study found no positive correlation between probiotics and low mood, but additional research on probiotics' role in mental health conditions was encouraged [43]. 

Gut dysbiosis is common with acute mental illness and chronic fatigue [44]. It is also postulated that synbiotic supplementation is an adjunct treatment for moderate depression. The study highlighted synbiotic treatment as a favorable adjunct therapy with fluoxetine to treat moderate major depressive disorder [45]. In another study on patients with major depressive disorder, patients reported a significant decrease in Beck Depression Inventory (BDI) scores in the probiotic group compared with the placebo. In contrast, the prebiotic group showed some improvement, but not statistically significant as in the probiotic group [46]. Talbott et al. demonstrated a close relationship and positive effect between probiotics, gut-brain axis, and mental well-being. The study examined probiotic supplementation in altering microbiome ecology and its impact on psychological mood states. Improved scores in positive and negative moods were observed for participants in the supplement group [47].

Furthermore, the supplement group also exhibited higher levels of good or diverse bacteria in the gut compared to the placebo group [47]. Psychobiotics are reportedly well tolerated and safe to improve symptoms of depression, anxiety, anhedonia, and sleep quality [48]. Similarly, Saccarello et al. report probiotics were safe in managing mild-to-moderate depression, improving depression, anxiety, and cognitive and somatic symptoms [49].

Several studies have emphasized and explained the domino effect of gut inflammation and depression [30]. A randomized controlled trial observed the impact of probiotics added with vitamin B7 in the diet of 82 depressed inpatients. One group of inpatients received a probiotic supplement containing different lactobacillus strains and bifidobacteria with vitamin B7. In contrast, the placebo group received vitamin B7, fish collagen, common horsetail plant supplement/extracts, and keratin matrix. Both groups showed improved psychiatric symptoms; however, no significant differences in outcomes were observed between supplemented group and the placebo group, suggesting that discrepancies could only be noted in the microbial diversity profile of the probiotic group. As the microbial diversity profile was increased in the probiotic group, probiotic supplements can be used as an adjunct therapy to balance the microbiota and maintain the homeostasis of the different pathways involved in the microbiota-gut-brain axis [50]. The RISTOMED (an internet-based project initiated for the elderly to prevent gut disturbances) study evaluated the effect of inflammation status on health-related quality of life, symptoms of anxiety, and depression in a healthy older population, as compared to group A (healthy diet), group B (probiotic with a healthy diet), group C (healthy diet plus monoterpene), and group D (healthy diet plus argan oil). The study reported a decrease in depressive symptoms in all four groups, which is attributable to a healthy diet, but found no associations between inflammation status and anxiety and depressive symptoms [51]. Another study suggested PROVIT (probiotics and vitamins) as an efficient adjunct therapy that could decrease inflammation and manage symptoms based on reductions in depression scores and IL-6 gene expression levels [52].

BDNF genes have been accepted for their role in the brain's plasticity and are linked with psychiatric illness [53]. The relationship between probiotics and depressive symptoms and their effects on BDNF levels have been explored in a study on 110 patients with low-to-moderate depression taking prebiotics (galactalogosaccharid), probiotics (*Lactobacillus helveticus *and *Bifidobacterium longum*), or placebo for eight weeks. The administration of psychobiotics showed increased BDNF levels and lower depression scores post-intervention, suggesting that probiotics have an effect in increasing BDNF levels, which modulates depressive symptoms [54].

Another randomized controlled trial by Schneider et al. in 2023, studying change in cognitive impairments in patients with depression, revealed improved immediate recall on the California Verbal Learning Test. The study, therefore, suggests that probiotics affect regulating the HPA axis and, consequently, hippocampal functioning. However, in line with the previously mentioned studies, there was no significant difference in the BDNF levels of the probiotic group, which depicts a discrepancy in generalizability [55].

Moreover, neurobiological abnormalities are observed in brain structure and function in the fronto-limbic network. Key observations were noted for the uncinate fasciculus (UF), connecting parts of the limbic system such as the temporal pole, subcallosal cortex, cingulate gyrus, and amygdala. A four-week placebo-controlled intervention reported changes in the right UF, suggesting probiotics to prevent neurodegeneration and a simultaneous improvement in depressive symptoms. Interactions were also observed in the subcallosal cortex, left orbitofrontal cortex (OFC), left hippocampus, and right amygdala [56].

However, the study by Yamanbaeva et al. states that divergent results are also reported, and the findings need to be explored more by involving a larger sample size [57].

Depression and Anxiety

Depression and anxiety co-occur in patients with IBS improving chronic idiopathic constipation [58]. A systematic review and meta-analysis support using probiotics for IBS when combined with other probiotic compounds [59]. Another study has shown positive effects in decreasing depressive symptoms in patients taking probiotics, although no substantial effect was observed on anxiety and IBS symptoms [60]. The prevalence of depression and anxiety symptoms in patients with chronic fatigue syndrome has been established, and probiotics have shown a significant decrease in anxiety scores, although no significant improvement in depressive scores. Alternations in neurotransmitters such as serotonin and dopamine were reported to be linked with depression and anxiety [61]. The diverse nature of the microbiota can help treat mental disorders, and recently fecal microbiota transplant has gained attention in treating psychiatric illness [62]. A study on 79 stressed adults evaluated the effects of probiotics in alleviating anxiety and stress symptoms. A much more diverse microbiota was found in the probiotic group. Although resilience to stress was present in both groups, it was found that gut-brain health is maintained not only by a diverse microbiota but also by some specific probiotic strains of *Lactobacillus*, *Bifidobacterium*, and *Faecalibacterium* [63]. Another study suggested probiotics as a feasible and natural intervention with anti-inflammatory properties and improvement in stress and anxiety symptoms, enhancing memory and cognition [64].

Chahwan et al. studied 71 participants with depressive symptoms and assessed differences in microbiota in these people after treatment with probiotics. The post-intervention probiotic group had lower depressive symptoms on the BDI scale than the placebo. A lower dysfunctional attitude score was reported in the probiotic group in subjects with less moderate depression. In contrast, no effect was seen in people with severe depression and those taking a placebo. The study suggested that while the microbiota diversity and composition were similar in both groups, the bacteria, Ruminococcus gnavus was found to be lower in the depressive group, suggesting its role in depression [65,66].

Another study measured stress, depression, and anxiety in 74 students preparing for the examination and found improved psychological symptoms and lower cortisol levels in the probiotic group. It was concluded that multiple-strain probiotics could help lower stress levels [67]. A similar pilot study examined the role of probiotics in reducing stress, anxiety, and depression in 40 students after one-week supplementation. The results did not demonstrate any substantial difference in the probiotic and placebo groups. Moreover, the intensity and severity of the symptoms were reduced by a few points, which suggests that intervention for one week was not substantial to make any conclusions [68].

Negative Studies and Critiques 

Psychobiotics have been restricted by the World Gastroenterology Organization and may harm people with underlying health issues and a weak immune system [69]. In a double-blind placebo-controlled study, the probiotic group performed slightly poorly in two measures studying memory, which the researchers attributed to chance. However, further assessment is needed in detail [70]. Probiotics may have value in treating depression and anxiety but are not yet approved by the FDA [71]. Another study found that supplementation of probiotics such as *Lactobacillus* in acute-onset GI problems yielded no response in children aged three months to four years [72]. Another study reports persistent intake of bacterial metabolite during developmental years may increase anxiety [73].

The probiotic use may be related to bacterial thickening in the small intestine. It can further lead to abdominal bloating, gas, pain, poor judgment, impaired short-term memory, concentration difficulties, and mental confusion (referred to as brain fog) [74]. This research concluded that the symptoms improved as the effect of the probiotics altered with antibiotics. 

The details of the studies included in the review are summarized in Table 1.

**Table 1:** Summary of studies evaluating the effects of probiotics on mental disorders. BAI, Beck Anxiety Inventory; BCAA, branched chain amino acids; BDI, Beck Depression Inventory score; BDNF, brain-derived neurotrophic factor; BUT, Body Uneasiness Test; CAN-BIND, Canadian Biomarker Integration Network in Depression; CANTAB Battery, Cambridge Neuropsychological Test Automated Battery; CES-D, Centre for Epidemiological Studies - Depression Scale; CFS, chronic fatigue syndrome; CRF, corticotropin-releasing factor; DASS, Depression Anxiety and Stress Scale; DSF, De Simone formulation; EWL, Eigenschaftswörterliste; FFQ, Food Frequency Questionnaire; FPG, fasting plasma glucose; GAD-7, Generalized Anxiety Disorder; GI, gastrointestinal; GOS, Galactooligosaccharide; HADS-A, Hospital Anxiety and Depression Scale-Anxiety; Gsh, glutathione; HADS-D, Hospital Anxiety and Depression Scale-Depression; HRQoL, Health-Related Quality of Life; HAM-A, Hamilton Anxiety Rating scale ; hs-CRP, high-sensitivity c-reactive protein; IBS, irritable bowel syndrome; iCGI, Improved Clinical Global Impression; IL, interleukin; KEGG, Kyoto Encylopedia of Genes and Genomes; LEIDS-r, Leiden Index of Depression Sensitivity – Revised; MADRS, Montgomery-Åsberg Depression Rating Scale; MDD, major depressive disorder; MCS-HRQol, Mental Component Score - Health-Related Quality of Life; MINI, Mini International Neuropsychiatric Interview; NFKB1, nuclear factor kappa B subunit 1; POMS, Profile of Mood States Scale; PSQI, Pittsburgh Sleep Quality Index; PSS, perceived stress scale; QIDS-SR16, Quick Inventory of Depressive Symptomatology – 16 items; SAMe, S-adenosylmethionine; SCL-90-R, Symptom Checklist-90-Revised; SECPT, Socially Evaluated Cold Pressor Test; SHAPS, Snaith-Hamilton Pleasure Scale; SSS, Symptom Severity Score; STAI, State Trait Anxiety Inventory; TAU, treatment as usual; TNF, tumor necrosis factor; VAS, visual analogue scale; VLMT, Verbal Learning Memory Test; Z-SDS, Zung Self-Rating Depression Scale

Author, Year	Aim	Participants	Intervention	Variables Measured in the Study	Results
Gruenwald et al. (2002) [31]	Evaluating effects of probiotic multivitamin on stress and immune function	42 adults experiencing moderate-to-intense burnout and stress	6-month intervention: multivitamin probiotic capsule: *Lactobacillus acidophilus, Bifidobacterium bifidum*,* Bifidobacterium longum*	Stress/exhaustion reduction (EWL Questionnaire), GI disturbances, and number of infections	Reduction in infections and GI disturbances. Improved positive condition and reduced negative condition. Overall, general condition enhanced.
Marcos et al. (2004) [33]	Evaluating effect on the immune system and anxiety by milk containing probiotics in university students	155 college students facing examination stress	6-week intervention: 200 mL of fermented milk containing* Lactobacillus delbrueckii *subsp. *bulgaricus*, *Streptococcus* subsp. *salivarius thermophilus*, and *L. casei*; or semi-skimmed milk of 200 mL	(State Trait Anxiety Inventory anxiety questionnaire) , blood hematology, lymphocyte production, cytokine production, serum cortisol, blood biochemistry	Rise in lymphocyte number and stable CD-56 immune cell count in the probiotic group. Anxiety levels increased in both groups between baseline and end results.
Diop et al. (2008) [34]	Assessing the effects of probiotics on psychological and GI symptoms caused by stress	75 volunteers with stress symptoms	3-week intervention: probiotics containing *L. acidophilus* and *B. longum*; or placebo	GI and psychological symptoms caused by stress	Psychological symptoms did not improve. Stress-induced symptoms such as abdominal pain and vomiting significantly reduced post-consumption of probiotics.
Berding et al. (2021) [35]	Determining the effect of probiotic supplement consumption on cognitive performance and acute stress	18 healthy female participants	12-week intervention: polydextrose or maltodextrin	CANTAB Battery, SECPT, Cohen’s PSS, BDI-II, HADS-A and HADS-D psychopathological symptoms (SCL-90-R); FFQ; GI symptom VAS	Improvement with polydextrose supplement in cognitive performance
Yang et al. (2016) [36]	Effects of probiotic supplementation in reducing pre- surgical anxiety in laryngeal cancer patients	30 laryngeal cancer patients with pre-surgery anxiety; 20 healthy volunteers	3-week intervention: probiotic containing *Clostridium butyricum*; or placebo	HAM-A scale, CRF levels, heartbeat check at morning and evening during waiting period for surgery	Anxiety scores significantly reduced in the probiotic group before surgery. There were steady heartbeat levels in the prebiotic group, whereas the placebo group showed risen heartbeat during surgery wait period. CRF levels were stable in the probiotic group, whereas they kept fluctuating in the placebo group.
Gualtieri et al. (2020) [40]	Determining the effect of psychobiotics in mainlining the allele A of IL-1β gene, which is the carrier of cytokines/psychological distress	97 subjects	12-week intervention: probiotic oral suspension group or placebo control group	HAM-A, BUT, and SCL-90-Revised, genotyping - DNA extracted from salivary samples	Probiotics mitigates anxiety symptoms.
Akkasheh et al. (2016) [42]	Assessing the effects of probiotics on metabolic status and depressive symptoms in MDD patients	40 patients with MDD	8-week intervention: Probiotics containing *L. acidophilus*,* L. casei*, and *B. longum*; or placebo	BDI for depressed mood, insulin metabolism, FPG, lipid concentrations, GSH levels, hs-CRP, antioxidant capacity	Significant reduction in BDI scores, oxidative damage, serum insulin levels, and hs-CRP levels. Gsh levels were increased and there was greater insulin resistance.
Romijn et al. (2017) [43]	Efficacy of probiotics as main treatment in individuals with low mood	79 adults with depression (mild to moderate)	8-week intervention: probiotics containing *L. helveticus*; or placebo	Depression and anxiety symptoms (DASS-42, iCGI, QIDS-SR, MADRS), severity (GAF), GI disturbance symptoms (IBS-SSS), hs-CRP, gene levels (IL-1β, IL-6) TNF-α, vitamin D, BDNF	Probiotic intervention increased vitamin D levels, improving mood. However, it did not affect other psychological symptoms and blood biomarkers. IBS symptoms were more severe in the probiotics group as compared with placebo.
Ghorbani et al. (2018) [45]	Effectiveness of synbiotic supplementation in treating moderate depression	40 outpatient adults with clinically diagnosed moderate depression	6-week intervention: synbiotic capsule and fluoxetine; and placebo capsule and fluoxetine	depression score (HAM-D)	Significant reduction in HAM- D scores of the synbiotic group.
Kazemi et al. (2019) [46]	Comparing the effects of prebiotics and probiotics supplementation on symptoms of depression and tryptophan metabolism	110 adults with depression (mild to moderate)	8-week intervention: Probiotics (*L. helveticus *and *B. longum*); or prebiotics includes GOS; or placebo	Depression intensity (BDI), tryptophan ratio, serum tryptophan/BCAAs ratio	Probiotics decreased BDI score and tryptophan ratio. Prebiotics significantly reduced tryptophan/branch chain amino acids ratio
Talbott et al. (2019) [47]	Evaluating effects of synbiotic supplementation in microbiome changes and mood states	32 individuals with "eustress"	1-month intervention: synbiotic supplement (*L. helveticus* R0052, *B. longum* R0175, *L. rhamnosus *R0011, GOS, and phytonutrients)	Psychological factors affecting mood state: POMS	Good bacteria (*Lactobacillus *and *Bifidobacterium*) increased in the microbiota; decrease in subscales of tension, depression, fatigue, and confusion; global mood score improved.
Wallace et al. (2021) [48]	Determining the efficacy, safety, and tolerability of probiotics on depression	10 participants	8 week intervention: *L. helveticus *R0052 and *B. longum *R0175	CAN-BIND, MADRS, QIDS-SR16, SHAPS, GAD-7, STAI, PSQI	Probiotics helped in alleviating the symptoms of depression in treatment-naïve and moderately depressed patients. Also, supported that probiotics are safe and well-tolerated in the population.
Saccarello et al. (2020) [49]	Assessing the effects of a combination of probiotics in mild-to-moderate symptoms of depression	90 patients with mild-to-moderate depression	6-week intervention: SAMe and *Lactobacillus plantarum* HEAL9 1 × 10⁹ CFU; or placebo	Z-SDS	Reduction in Z-SDS scores after 2 weeks of intervention; improved symptoms of anxiety, depression, and cognitive and somatic components.
Reininghaus et al. (2020) [50]	Evaluating the effect of supplementary probiotic treatment and vitamin B7 in depression	82 individuals with depression	28 days intervention: multistrain probiotic plus biotin treatment; or placebo plus biotin treatment	Microbiome samples, KEGG analysis	A 4-week probiotic plus biotin treatment inpatient individuals with MDD showed an improved overall effect in clinical treatment, whereas the placebo group only differed in microbial diversity profile.
Bourdel-Marchasson et al. (2020) [51]	Determining effect of combining healthy diet, probiotics, and argan oil in decreasing depressive symptoms	125 older subjects	8-week intervention: RISTOMED diet alone, or RISTOMED diet + DSF probiotic blend, or RISTOMED diet + AISA−5203-L orange peel extracted monoterpene d-limonene, or RISTOMED diet + Native® Argan oil	Inflammatory parameters, mental and physical components, HRQol, anxiety symptoms (STAI), and depressive symptoms (CES-D)	MCS-HRQoL improved in the first and third intervention groups. There was a decrease in depressive symptoms.
Reiter et al. (2020) [52]	Determining gene expression change in MDD patients after multispecies probiotic supplements	61 inpatients with MDD	4-week intervention: multispecies probiotic or placebo	Effects on gene expression of TNF, NFKB1, and IL-6	Intervention group showed decreasing IL-6 gene expression.
Heidarzadeh-Rad et al. (2020) [54]	Determining the effect of psychobiotic supplement on serum BDNF levels in depressive patients	110 patients with low-to-moderate depression	8-week intervention: *B. longum* and* L. helveticus* and excipients; or placebo containing only excipients	BDNF levels, BDI	Improvement in depressive symptoms with *B. longum* and *L. helveticus* possibly by increasing BDNF levels.
Schneider et al. (2023) [55]	Investigating the effects of probiotic supplementation on cognitive symptoms in depression	43 participants with MDD	4-week intervention: probiotic group (19 subjects) and placebo group (12 subjects)	VLMT, Corsi Block Tapping Test, Trail Making Test, and BDNF levels	Improved affective symptoms and verbal episodic memory. Balancing cognitive impairments and altered hippocampal activity.
Yamanbaeva et al. (2023) [57]	Assessing the effects of probiotics supplementation for four weeks on symptoms of depression by investigating fronto-limbic structure, function, and perfusion	32 inpatients with depressive episodes	4-week intervention: probiotic oral suspension consisting *Streptococcus thermophilus*, *Bifidobacterium infantis*, *L. acidophilus*, *L. plantarum*, *Lactobacillus paracasei*, *Lactobacillus delbrueckii*; and placebo control group with TAU	Diffusion tensor imaging, resting state functional MRI, arterial spin labeling, HAM-D	Probiotics affected brain functions and structure in the fronto-limbic network, which are relatively reduced during depressive episodes.
Pinto-Sanchez et al. (2017) [60]	Assessing effects of supplementation of *B. longum* bacteria in IBS patients with anxiety and depression	44 IBS patients with depressive symptoms (mild to moderate)	6-week intervention: powdered probiotics (*B. longum*); or placebo	Anxiety and depression score (HAD), IBS symptoms, somatization, quality of life, brain activation changes, inflammatory biomarkers, neurotransmitter levels, and stool and urine microbiota population	Probiotics intake reduced HAD scores and depression levels significantly, quality-of-life scores improved, and there was no effect on anxiety and IBS symptoms. Probiotics lowered negative emotional stimuli responses; placebo and probiotic groups had similar levels of inflammatory biomarkers, stool microbiota, and neurotransmitters.
Ma et al. (2021) [63]	Determining probiotic supplements in alleviating stress and anxiety symptoms	79 subjects (follow-up study)	12-week intervention: *L. plantarum* P8 or placebo	Shannon diversity index	There was a positive association between probiotic-induced gut-microbiota modulation and stress/anxiety alleviation in stressed adults.
Lew et al. (2019) [64]	Assessing the effect of probiotic *L. plantarum *P8 in alleviating stress and anxiety and enhancing memory and cognition	103 adults with stress	12-weeks intervention: *L. plantarum *P8 or placebo	DASS-42 questionnaire, plasma cortisol levels	There was alleviation of selected stress, anxiety, and improved memory and cognitive symptoms such as social-emotional cognition and verbal learning.
Chahwan et al. (2019) [65]	Assessing probiotic supplementation on depressive symptoms	71 participants with depression (mild to severe)	8-week intervention: probiotics sachet* (B. biﬁdum, B. lactis, L. acidophilus, L. brevis, L. casei, L. salivarius*, and *Lactococcus lactis*) or placebo	Depression and anxiety scores (MINI; BDI; DASS-21; LEIDS-r, BAI) stool sample analysis, dietary assessments, client satisfaction questionnaire	Significant reduction in cognitive reactivity of the probiotic group compared with placebo. No appreciable difference in microbiota profiles and depression and anxiety scores.
Venkataraman et al. ( 2021) [67]	Determine effects of multi-strain probiotic supplements on students facing examination stress	80 students (18-24 years), but 74 completed	28-day intervention: *Bacillus coagulans* Unique IS2, *Lactobacillus rhamnosus* UBLR58, *Bifidobacterium lactis *UBBLa70, *L. plantarum *UBLP40 (each of 2 billion CFU); *Bifidobacterium breve* UBBr01, *Bifidobacterium infantis* UBBI01 (each of 1 billion CFU) capsule with glutamine (250 mg) twice a day	PSS, DASS, and STAI	The multi-strain probiotic is effective in reducing examination-associated stress.
Seigel et al. (2020) [68]	Determining reduction in stress, anxiety, and depressive symptoms with *B. longum* intake	84 students	1-week intervention: *B. longum *or placebo	PSS, CES-D, and STAI Y2 FORM	7 days of intervention did not reduce stress, depressive symptoms, or anxiety in generally healthy young adults.
Rao et al. (2009) [74]	Evaluating efficacy of probiotic administration in CFS patients with depression and anxiety symptoms.	35 patients with CFS	8-week intervention: probiotics (*L. casei*) or placebo	Depression and anxiety scores (BDI, BAI), profile of stool microbiota	*Lactobacillus *and *Biﬁdobacterium* fecal levels increased in the probiotic group with a significant improvement in anxiety symptoms, However, no change was seen in depression scores.

## Conclusions

The quest for alternative, effective, accessible, safe treatment options for serious mental health conditions has been the focus of research in the last few decades. The knowledge about the gut-brain axis has been expanded, and its effects on mental health have been empirically established and widely accepted. In recent times, the role of probiotics in mental health has been both a matter of debate and controversy. Although the studies in the review suggest that probiotics may be an effective measure to treat mental health disorders, most of the studies lacked power and had weak study designs; therefore, results could not be generalized to a wider population, and the evidence remains inconclusive. It has been marketed as a well-tolerated safe alternative with a positive effect on mental health conditions via its effects on the gut microbiome. However, due to the shorter duration of studies, its long-term efficacy and risks are yet to be investigated. The specificity of the strain and quantities of prescribed probiotics also remain crucial. Studies have also advised restricting their use in patients with weak immune systems and underlying serious conditions. It has been largely marketed as a cost-effective measure, although given the overall hidden cost of mental health and push from the unregulated food supplements industry, there is a need for a more robust cost-benefit analysis to support these claims. The evidence in support of psychobiotics is limited but not without risks including lack of response, worsening of the illness, and the costs associated with these supplements. A consultation with an informed healthcare provider is recommended to understand the risk-and-benefit rationale of the empirical studies. A regular follow-up to ascertain the response to complementary treatment under the close supervision of a physician is recommended.
